# Ecosystem sentinels for climate change? Evidence of wetland cover changes over the last 30 years in the tropical Andes

**DOI:** 10.1371/journal.pone.0175814

**Published:** 2017-05-24

**Authors:** Olivier Dangles, Antoine Rabatel, Martin Kraemer, Gabriel Zeballos, Alvaro Soruco, Dean Jacobsen, Fabien Anthelme

**Affiliations:** 1 Institut de Recherche pour le Développement (IRD), EGCE, Gif-sur-Yvette, France and Université Paris-Sud, Orsay, France; 2 Pontificia Universidad Católica del Ecuador, Facultad de Ciencias Exactas y Naturales, Quito, Ecuador; 3 Unidad de Limnología, Instituto de Ecología, Universidad Mayor San Andrés, La Paz, Bolivia; 4 Université Grenoble Alpes, CNRS, IRD, Institut des Géosciences de l’Environnement (IGE), Grenoble, France; 5 Escuela Militar de Ingeniería, Carrera de Ingeniería Geográfica, Bajo Irpavi, La Paz, Bolivia; 6 Department of Geography, Byrd Polar and Climate Research Center, Ohio State University, Columbus, Ohio, United States of America; 7 Instituto de Investigaciones Geológicas y del Medio Ambiente (IGEMA), Campus Universitario UMSA; 8 Freshwater Biological Laboratory, Biology Department, University of Copenhagen, Copenhagen, Denmark; 9 AMAP, IRD, CIRAD, CNRS, INRA, Université Montpellier, France; Consejo Superior de Investigaciones Cientificas, SPAIN

## Abstract

While the impacts of climate change on individual species and communities have been well documented there is little evidence on climate-mediated changes for entire ecosystems. Pristine alpine environments can provide unique insights into natural, physical and ecological response to climate change yet broad scale and long-term studies on these potential ‘ecosystem sentinels’ are scarce. We addressed this issue by examining cover changes of 1689 high-elevation wetlands (temporarily or perennial water-saturated grounds) in the Bolivian Cordillera Real, a region that has experienced significant warming and glacier melting over the last 30 years. We combined high spatial resolution satellite images from PLEIADES with the long-term images archive from LANDSAT to 1) examine environmental factors (e.g., glacier cover, wetland and watershed size) that affected wetland cover changes, and 2) identify wetlands’ features that affect their vulnerability (using habitat drying as a proxy) in the face of climate change. Over the (1984–2011) period, our data showed an increasing trend in the mean wetland total area and number, mainly related to the appearance of wet grassland patches during the wetter years. Wetland cover also showed high inter-annual variability and their area for a given year was positively correlated to precipitation intensities in the three months prior to the image date. Also, round wetlands located in highly glacierized catchments were less prone to drying, while relatively small wetlands with irregularly shaped contours suffered the highest rates of drying over the last three decades. High Andean wetlands can therefore be considered as ecosystem sentinels for climate change, as they seem sensitive to glacier melting. Beyond the specific focus of this study, our work illustrates how satellite-based monitoring of ecosystem sentinels can help filling the lack of information on the ecological consequences of current and changing climate conditions, a common and crucial issue especially in less-developed countries.

## Introduction

Climate change is a major challenge for species and ecosystems, resulting in significant modifications in biodiversity patterns and processes worldwide [1, 2]. One promising approach to study the integrated effects of climate change on biodiversity and on ecosystem services involves monitoring the responses of entire ecosystems, as they may represent sensitive indicators of both drivers and responses [3]. As the overall distribution of ecosystems largely reflects current climate conditions, it is likely that changes in temperature, precipitation, and carbon dioxide levels, together with additional environmental changes (e.g., grazing intensity, glacier melting, invasive species) would significantly alter the geographical distribution of ecosystems [4]. Furthermore, rapid and widespread modifications in the geographical distribution of entire ecosystems may deeply impact biogeochemical cycles (e.g., water and carbon) at both local and regional levels [5]. Climate change is also expected to alter significantly the quality and the quantity of natural resources to human societies [6].

Ecosystem sentinels for global changes have already been identified for several biomes throughout the world such as savannas [7] and coral reefs [8]. However, in most cases, it remains difficult to disentangle climate change effects from anthropogenic pressures. Ideally, ecosystem sentinels should present both a high sensitivity to climate change and a low one to human perturbation so that it is possible to identify early signals of climate change and variability while minimizing the number of confounding factors potentially masking its detection [9]. Pristine arctic and alpine environments such as aquatic systems (e.g., lakes and rivers, [10, 11, 12]) and alpine grasslands (e.g., [13]) can provide quite unique insights into natural, physical and ecological response to climate change as many arctic and alpine communities are located near the edge of their climatic limits [14]. While these ecosystems have been identified since the late 1990’s as ideal candidates to set up environmental observatories of the ecological effects of climate change (e.g., The Global Observation Research Initiative in Alpine environments—[15]), the lack of historical data in such remote and understudied regions has not allowed to obtain information on how entire natural communities and ecosystems have responded to recent patterns in climate change [16].

**Fig 1:**
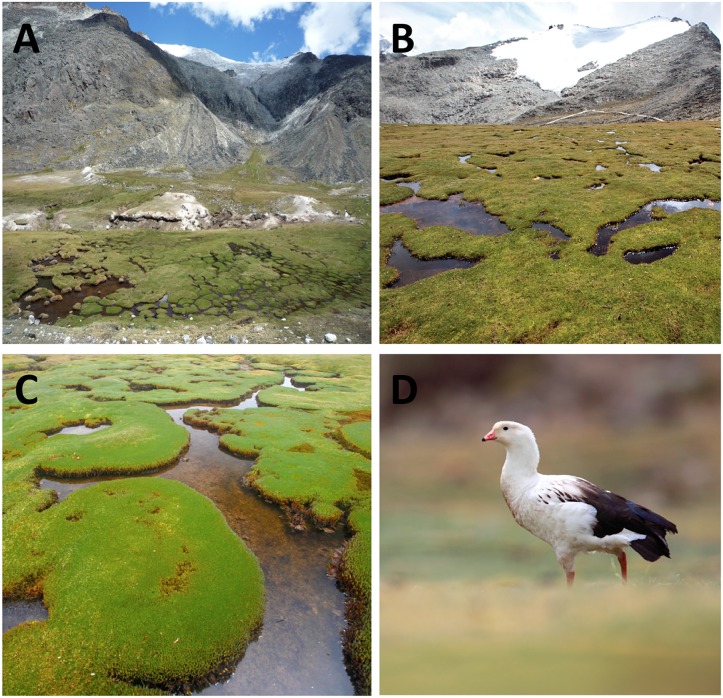
Photographs of the study system in the Cordillera Real of Bolivia (above 4500 m). A. Wetland in the arid landscape of the puna, B. Proximity of wetlands and glaciers, C. Cushion plants (*Distichia muscoides*) composing the wetlands, D. Example of a bird species (*Chloephaga melanoptera*) tightly dependent on wetlands. © Fabien Anthelme (A) and Olivier Dangles (B-D).

In the cordilleras of the tropical Andes, high-elevation wetlands (over 4500 m above sea level, a.s.l.) are located in environments of relatively low annual precipitation and soil moisture deficits within a region also known as *puna* [17]. These wetlands can be described as areas of swamp, marsh, meadow, fen or peatlands, perennial or temporary, with water that is stagnant or flowing (Fig 1A). These ecosystems may represent ideal ecosystem sentinels of the effects of recent climate change for several reasons (see also [18]). First, most of these wetlands are located in topographical depressions either downstream from glaciers that are rapidly receding ([19], Fig 1A and 1B) or within small watersheds presenting a strong coupling between atmospheric forcing (precipitation patterns) and water flows [20]. The contribution of glaciers to water input may account for as much as 27% during the dry season [21], explaining why their recession/disappearance may affect adjacent wetlands. Second, high-elevation wetlands in the Andes provide important biogeochemical functions such as water flow regulation and organic matter accumulation [22]. Their functioning can be easily affected by the change of climatic conditions as a simultaneous increase in temperature and decrease in water level may favor soil oxygenation, organic matter decomposition and, in turn, the release of large amounts of CO_2_ into the atmosphere within only a few years [23, 24]. There is experimental evidence that persistently high temperatures can reduce carbon accumulation in peatlands by up to 30% owing to the decline of plant productivity and the rise of microbial decomposition of organic matter [25]. In the high Andes, a 3°C increase in temperature could result in a theoretical 600-m upward movement of plant species [26], with potential impacts on wetland flora composition. All this can have potentially significant consequences for carbon storage/release dynamics [27] and wetland productivity that maintains an extensive camelid pastoralism supporting over 700,000 rural Andean households in the region [28, 29]. Third, being “wetlands in drylands”, Andean high-elevation wetlands can be easily detected and delimited using satellite images. Indeed, optical satellite data (e.g., LANDSAT) provide quite large time series (30 years) with an access to near infrared bands, which is effective to assess vegetation density [30].

In this study, we used a combination of high-resolution satellite images (PLEIADES) and the LANDSAT archive to map the location and extent of wetlands across the Cordillera Real of Bolivia, and to quantify changes in their surface area over the last 30 years. As high-altitude wetlands in the Bolivian Andes are almost entirely covered by vegetation (see Fig 1), our satellite image analyses mainly documented changes in vegetation cover and dynamics over time. Vegetation dynamics finely responds to changes in water and soil conditions, in turn influenced by climate change. The two specific objectives of this study were 1) to identify environmental factors (e.g., glacier cover in the catchment, wetland size, and watershed size) that affect wetland long-term surface changes at both regional and individual scales, and 2) to identify the characteristics of individual wetlands (e.g., shape, size, and location in the catchment) that control their propensity to dry (as a result of changes in abiotic conditions). This latter objective intends to allow developing a vulnerability map of high altitude wetlands to guide conservation plan.

## Materials and methods

### Study region and climate settings

**Fig 2:**
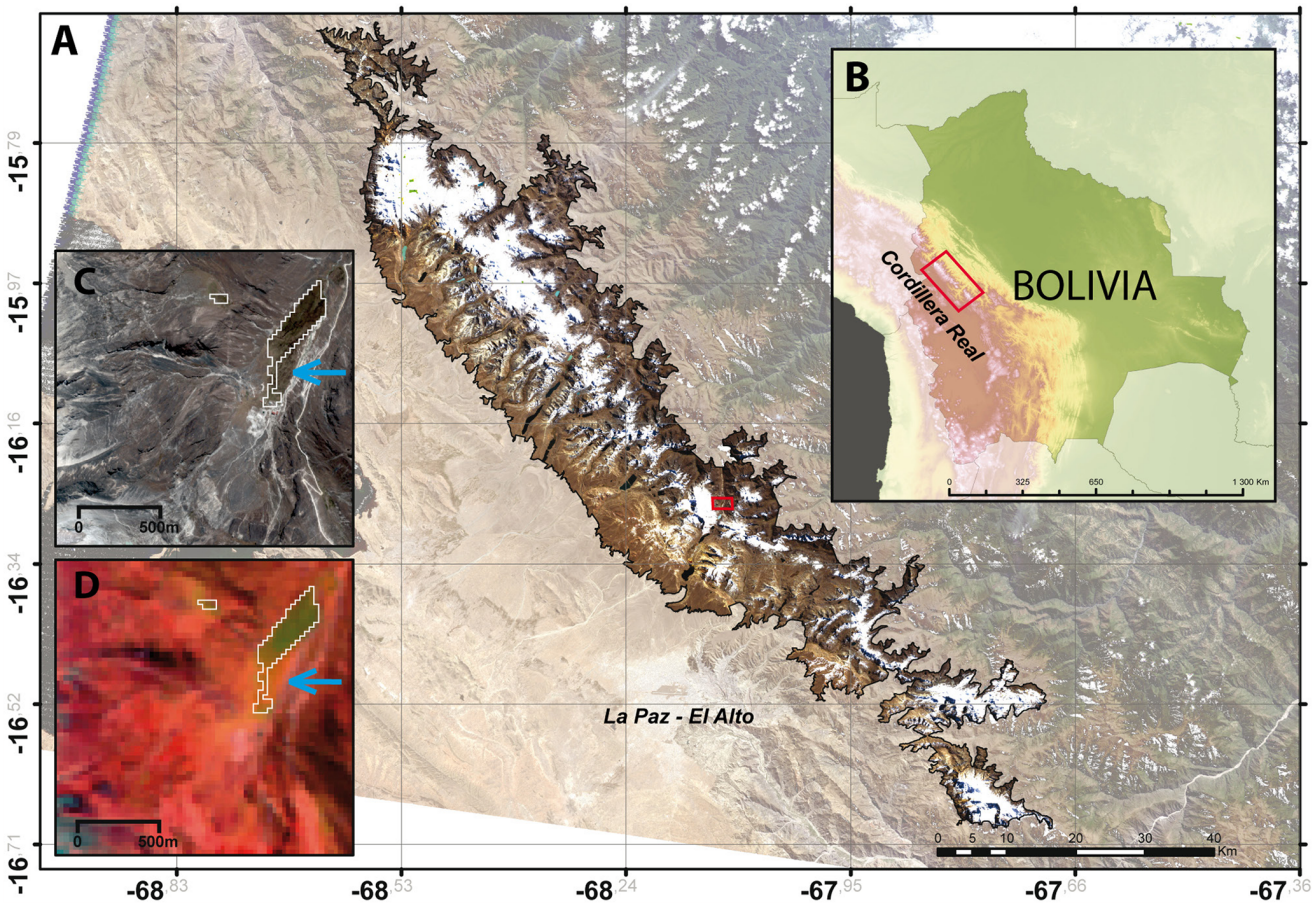
Map of the study site and satellite images. Map of the study area in the Cordillera Real, Bolivia (A, B) and examples of high elevation wetland delineation from Pléiades image recorded in 2013 (C) and Landsat-TM 2007 (D). The white outlines in (D) show the extension of the wetlands quantified from the 2007 Landsat image. Overlapping these outlines on the 2013 Pléiades image (C) shows the overall good agreement with the extension of the wetland six years later. The blue arrow illustrates the growth of the wetland between the two dates.

The Cordillera Real (15°45’– 16°45’ South, 67°40’– 68°40’ West) is part of the Bolivian Eastern Cordillera between the Altiplano plateau in the west and the Amazon basin in the east. The Cordillera Real stretches from south-south-east to north-north-west over 130 km with an approximate width of 15–20 km (Fig 2A and 2B). Several summits reach more than 6,000 m a.s.l.; however, due to the low latitude, glaciation is reduced (about 150 km^2^) and glaciers are relatively small: 80% cover less than 0.5 km^2^ [19, 21]. The study region is the upper part of the Cordillera Real, *i*.*e*. above 4500 m a.s.l. This elevation limit was chosen to ensure the exclusion of most human activities such as villages, mining activities, overgrazing, water collection and artificial lakes that may impact the observed ecosystems.

The climate of this tropical cordillera is characterized by a low annual temperature range (< 5°C) with a slight peak in summer (December–February) when solar radiation and air moisture are at their maximum. Incident solar radiation is strong all year round and not seasonally contrasted due to the low latitude and because at its maximum (in summer) it is attenuated by pronounced cloudiness. On the other hand, when incident solar radiation is minimum (May–August) the atmosphere is dry and cloudiness is not a limiting factor. In contrast, seasonality of humidity and precipitation is strong. Humid and precipitation fluxes come from the northeast via the Amazonian basin and precipitation can reach 800–1000 mm yr^−1^ on glaciers at about 5000 m a.s.l [31]. As a consequence, the eastern side of the Cordillera is more humid than the western side. The October–December period is characterized by a progressive increase in moisture and precipitation. Accumulation rates reach maximum between January and April (2/3 of the total amounts). The period between May and September is characterized by a dry atmosphere, strong westerly winds at high elevations, low cloudiness, and cooler temperatures. The seasonal pattern displays significant modifications due to climatic variability that is mainly controlled by El Niño Southern Oscillation (ENSO) phases at the multiannual time scale [32]. However, precipitation regimes in the southern region of the tropical Andean are moderately correlated with sea surface temperature anomalies with a ENSO–rainfall signal highly variable depending on latitude, elevation and the side of the Andes [33]. A review of climate changes in the 20^th^ century along the tropical Andes [34] reported 1) a decreasing trend in precipitation after the mid-20^th^ century in southern Peru and the Bolivian Altiplano [35, 36, 37]; 2) a rise of air temperature at an accelerated rate relative to the global average over recent decades. Mean air temperature has increased by 0.10°C decade^−1^ in the last 70 yr, which represents an overall temperature increase of 0.68°C since 1939 [35].

### High-elevation wetlands

While vegetation cover is generally sparse in the alpine belt of the cordillera (dominated by *Festuca dolichophylla* J. Presl, *Agrostis tolucensis* Kunth and various species of *Pycnophyllum* and *Deyeuxia*) several deep valleys formed by the last ice ages shelter extended wetlands whose characteristics vary depending on water availability. In our study, the term “wetlands” defines a range of ecosystems from temporary wet grasslands to perennial peat bogs. Most of these wetlands are restricted to the alpine and sub-alpine belts at elevations between 3200 to 5000 m a.s.l., and appear as green oases in an overall arid landscape (Fig 2C and 2D). Their vegetation contrasts sharply with surrounding terrestrial communities by having plant cover usually greater than 70% and high plant productivity [17]. These wetlands are characterized by the so-called cushion plants (Fig 1C), some compact, low growing, mat forming plants commonly found in alpine and polar environments around the world. In Bolivia, above 4000 m a.s.l., cushion plants of the Juncaceae and Cyperaceae families (in decreasing order of abundance: *Oxychloe andina* Phil., *Distichia muscoides* Nees and Meyen, *Phylloscirpus deserticola* (Phil.) Dhooge and Goetgh. and *D*. *filamentosa* Buchenau dominate the lawn and hummock communities while aquatic macrophytes (e.g., *Ranunculus uniflorus* Phil. ex Reiche, *Lilaeopsis macloviana* (Gand.) A.W. Hill, *Callitriche heteropoda* Engelm. ex Hegelm. are found in open water pools [38, 39]. High Andean wetlands in the Peruvian and Bolivian *puna* are important staging grounds for migratory birds ([40], Fig 1D) and breeding grounds for invertebrates, amphibians, and fish [17, 41]. The taxonomic diversity of this rich fauna is generally positively correlated with habitat area [40, 42]. Wetlands’ ability to promote vegetation growth lessens soil erosion and buffers water flow, providing a steady flow of water downstream while reducing the severity of floods and droughts [17]. They also support unique ecosystems and services that sustain the livelihoods of people by providing year-round water supply and forage for wild and domestic animals. A regional initiative for the conservation and wise use of high Andean wetlands was approved in November 2005 within the framework of the Intergovernmental Convention on Wetlands (RAMSAR, [43]). While much of this vegetation is perennial, part of these wetlands can dry out or extend on the margins, depending on precipitation intensity. Moreover, when fed by rains, parts of the mineral landscape can become vegetated by pioneer herbaceous species forming new grasslands.

### Satellite images acquisition and processing

To quantify the spatio-temporal changes in glaciers and wetlands at the scale of the Cordillera Real, images from the LANDSAT and PLEIADES satellites were used. The first step consisted in the collection, correction, treatment, and classification of the satellite images. From this information, the inventory, analysis and categorization of wetland variability patterns were conducted. The methodology is detailed below.

S1 Table presents the list of the used LANDSAT images covering the period 1984–2011 and the PLEIADES images from 2013. All the images were recorded during the dry season (between May and August) to ensure minimal snow-cover and cloud-cover. This period is also the most relevant for conservation and development issues as native fauna and livestock strongly depend upon wetland pastures during the dry season. Landsat images were selected for their radiometric characteristics and spatial resolution (15 m in panchromatic and 30 m in multispectral); such images are widely used for multi-temporal analysis of wetlands [30]. All LANDSAT images were downloaded from the Earth Explorer server of the United States Geological Survey (http://earthexplorer.usgs.gov/). The LANDSAT 7 ETM+ images after May 31, 2003 were dismissed as, from this date, these images present systematic stripes, due to a malfunction of the sensor (SLC-off). PLEIADES images were acquired for their high resolution (0.5 m) allowing complementary information to be accessible. The ASTER Global Digital Elevation Model version 2 (GDEM, http://gdem.ersdac.jspacesystems.or.jp/) was used to delineate watersheds and characterize their morphometric variables (see below).

All images were corrected and digitally processed in the same way. Before generating indexes and classification (see 3.3) the following operations were performed: 1) co-registration of images, 2) atmospheric correction, and 3) conversion of digital numbers to reflectance values. Co-registration was performed with the tool *AutoSync* of ERDAS IMAGINE v.2011 software. This tool algebraically recognizes the coordinates of common points between two satellite images and co-register them automatically using stereo-correlation between the two images. To carry out this process, a reference image is chosen to which all other images are geometrically corrected. In our case the LANDSAT 7 ETM+ from July 11, 2002 Band 8 was chosen as the reference image. The atmospheric correction process was performed with the tool *ATCOR3* (Atmospheric correction v.3 GEOSYSTEMS GmbH company) of ERDAS IMAGINE software. For this processing, the program requires using a DEM from which different topographic variables are calculated: the slope, the aspect, elevation and sky-view factor. In addition, the tool also considers the exact time of the image acquisition, which provides information about the zenith and azimuthal positions of the sun, and therefore the incidence angles during the image acquisition. The transformation of the satellite image pixels digital numbers to reflectance values was performed using the *LANDSAT Reflectance* tool of ERDAS IMAGINE software. To carry out this process, we used the radiometric sensor calibration parameters available in the metadata files for each satellite image. This process allows taking into account the inclination of the sun at the time of the image capture.

All images were classified by normalized difference indexes for vegetation and snow (NDVI, NDSI) that allows automatically identifying on satellite images different types of objects from changes in their own spectral characteristics in different bands [30, 44]. To determine the threshold of vegetation, we used the high spatial resolution PLEIADES images. Indeed, wetlands are easily recognizable at a 50-cm resolution. The comparison of the real-color image and a combination bands involving the near-infrared band allowed us to distinguish and then to manually delineate the wetlands outline on the PLEIADES images using a GIS (Fig 2C and 2D). For the LANDSAT images, to determine the NDVI threshold allowing us accessing to the most accurate delineation of a wetland, we relied on the fact that the area of the entity extracted by using a NDVI threshold value is a function of the threshold value (S1 Table). So there is a relationship between the surface areas determined for any entity on the LANDSAT image using a NDVI threshold and the surface area of the same entity delineated on the PLEIADES image. This allowed us defining the most accurate threshold that maximizes the correlation of the two surface areas. We used 33 wetlands and compared their surface area computed on the PLEIADES image and a LANDSAT images with different NDVI thresholds to obtain the used value of 0.468 (S1 Fig). The overall uncertainty on the wetlands surface area was computed as the horizontal uncertainty of the position of the margin of the polygon representing a wetland. This horizontal uncertainty was quantified by varying the NDVI threshold from 0.448 to 0.488. These values match an interval of +/- 0.02 on the used NDVI threshold value of 0.468 and allow conserving a low RMSE value when comparing the wetland delineation between the PLEIADES and LANDSAT images for 2011 (see Supp. Mat.). This uncertainty varies from one year to another from few km^2^ (considering the wetland total area) up to +/- 10 km^2^.

For the glaciers, the NDSI threshold was set to 0.4 by comparing the glacier outline automatically obtained with field measurements [19, 31]. To automate the image processing, a classification algorithm was generated; divided into three steps: 1) images corrections, 2) classification, and 3) topological control for the final categorization of objects. The algorithm allowed obtaining for each LANDSAT image used in the study a vector layer of the extent of the glacier/snow and wetlands present in the images. These layers can be overlaid to illustrate the spatio-temporal changes in the study variables. We then calculated the total area and number of wetlands, and the area of glaciers and snow-covered surfaces directly from each annual layer.

### Data analyses

#### Long-term trends of wetland area and number and environmental factors

We estimated the regional long-term trends in wetland surface area by fitting linear models allowing different slopes and intercepts to be computed (ordinary least squares). The R-value and the p-value of each slope coefficient were also calculated. Due to the unknown correlated structure of the model, these statistics aim to provide information of the fitted linear trend of each time series, rather than testing a hypothesis.

We selected six ecologically relevant factors for which we tested their significance in influencing the overall wetland area trend: 1) elevation (m a.s.l.), 2) orientation (west/east), 3) slope (degrees from horizontal), 4) initial wetland size (*i*.*e*. habitat extent, m^2^)—these factors were directly given by the ArcGIS contingency table -, 5) watershed area (m^2^) and 6) percentage of glacier cover in the catchment (%GCC). For these last two factors, watershed delimitation was realized using *Spatial Analyst* in ArcGIS 10.0. The drainage area for 121 previously selected points (outlets) was calculated on the basis of ASTER GDEM v2. The glacier outlines and watershed contours shape files were merged using ArcGIS 10.0. This enabled computing the percentage of the glacier cover in the watersheds, a commonly used index to estimate the potential influence of a glacier on an aquatic system (e.g., [45]).

We then used analysis of covariance (ANCOVA) to determine if statistically significant differences in wetland area temporal trends depended on these six factors: orientation (west *vs*. east), elevation, slope, glacier cover in the catchment, initial wetland size and watershed area. For the five latter variables we compared the first and the last quartiles of each wetland variable distribution (e.g., 25% largest *vs*. 25% smallest wetlands, 25% steepest *vs*. 25% flattest wetlands and so on).

#### Wetland area related to changes in precipitations and glacier and snow-covered surfaces

To assess whether changes in wetland area was related to change in precipitations, we used precipitation data obtained over the (1984–2011) period from a meteorological station located in the Zongo valley (16°15’ S, 68°10’ W, *i*.*e*. within the study area). It is noteworthy that this station is the only one within our study area covering the entire study period. We tested the cross-correlation between the wetland area at time t, and the monthly precipitation over 1 to 12 months before time t, using the correlation function estimation in R. The best correlation was obtained for a period of three months prior to image acquisition. Wetland areas were then plotted against monthly precipitation and glacier and snow-covered surfaces measured over the study period. The R-value and the p-value of each slope coefficient were also calculated. We then combined the two predictive variables into a single multiple linear regression.

#### Drying frequency and vulnerability of individual wetlands

As each wetland presents highly diverse features that may affect its response to environmental alteration, individual wetland monitoring is essential to understand spatio-temporal dynamics and define factors that drive these changes. In particular, we observed on the satellite images that many individual wetlands were prone to dry out partially. The appearance of dry patches resulted in a reduction of the overall surface of available high-quality habitat for flora and fauna and of the connectivity among irrigated patches. Wetland patch drying can occur at different spatial scales with a potentially strong impact on a spatially structured plant and animal populations, especially for small organisms with limited mobility. These constitute the bulk of alpine wetland biodiversity. Even limited drying of tenths of meters can have significant impacts on population dispersal patterns living in alpine ecosystems, as shown for amphibians [46], insects [47] and crustaceans [48]. This suggests that habitat loss during dryer years could be a major issue to understand the vulnerability of individual wetlands over time.

**Fig 3:**
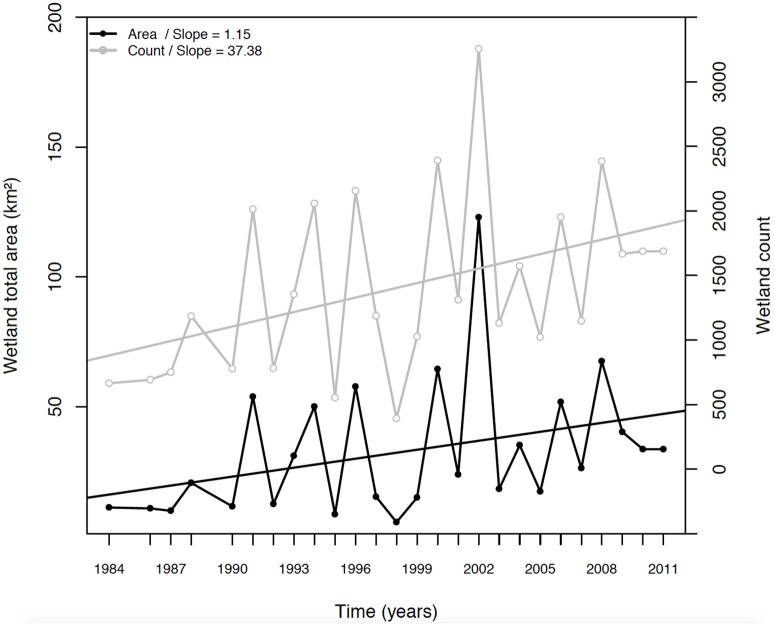
Changes in the total annual wetland surface area and wetland count over the 1984–2011 period in the Cordillera Real, Bolivia. The uncertainty in wetland cover calculations based on satellite images varies from a few km^2^ to 10 km^2^ depending on the year (see Methods).

Individual wetland monitoring cannot be automated in GIS and has to be performed manually. For this reason, of the 705 wetlands identified in 1984 in the study region, we randomly selected a sub-sample of 423 entities for this analysis. Drying events resulted in the division of wetlands in a certain number of fragments. For each year (between 1984 and 2011), a value was assigned for each fragment of one wetland entity thereby allowing counting the number of fragments resulting from drying since 1984. We recognize that using the mean of several years as a reference "wetland state" may have been more robust than a single year to perform our trend analyses, but it was technically impossible to run the "individual wetland monitoring" by using several years as a baseline. However, we consider that 1984 was an appropriate reference year, as it presented baseline values of wetland cover and number over the three decades (similar values were found in 1985, 1986, 1987, 1990, 1992, 1995, and 1998; see Fig 3).

Based on these analyses, we calculated the drying frequency of each individual wetland, *i*.*e*. the number of years over the 1984–2011 period for which several fragments were detected on the location of the reference wetland (S2 Fig). We also calculated the drying intensity, *i*.*e*. the average number of fragments resulting from drying, but as the two indices were highly correlated (> 0.67) we decided to present only the drying frequency.

We then used Generalized Linear Models (GLMs) to test the effects on wetland drying frequency of five variables used in the ANCOVA analysis (elevation, slope, wetland area, wetland watershed area, glacier cover in the watershed) and their interactions. A term ‘‘valley” was included to allow within-site comparisons, while controlling variations resulting from unmeasured valley-specific variables (*i*.*e*. hydrology, local climatic conditions). Valley was treated as a fixed effect, but we obtained similar results by means of the generalized linear mixed model function (glmmPQL; MASS library for R), with valley as random effect. To this list of seven factors we added another variable commonly used in habitat conservation studies: the shape of the wetland (see [49] for a recent review). The shape of a wetland may have significant influences on its functioning and response to climate change as highly convoluted shapes may be more susceptible to disturbances (e.g. drought): a proportionately greater amount of area is exposed to the dry matrix surrounding wetlands [50]. Following [51], we calculated a shape index (SI) of wetland units by dividing the unit perimeter by that of an equal-sized circle as follows:

$$SI=\frac{P}{2\sqrt{\pi A}}$$

where *P* is the perimeter of the wetland unit and *A* is wetland unit area. SI varies from 1 for perfect circles to 8 or higher for irregularly shaped fragments.

We then applied these results obtained with our sub-sample of 423 wetlands to our entire dataset (1689 wetlands in 2011) so that we could obtain a broad-scale and up-to-date prediction of their vulnerability status (see results for further information on our vulnerability index).

## Results

**Fig 4:**
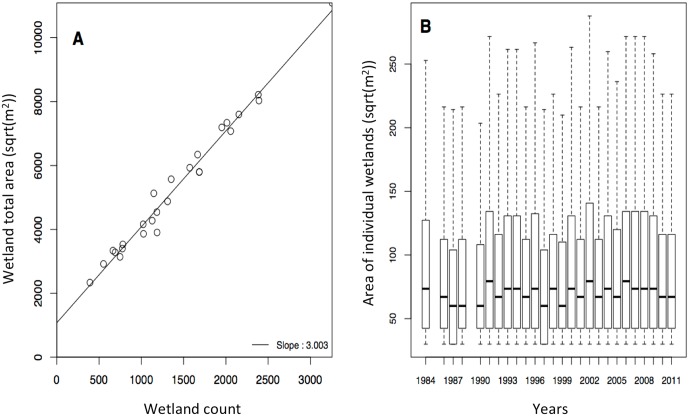
Area and number of wetlands. A) Relationship between high-elevation wetland surface area and count and B) changes in the distribution of individual high-elevation wetland surface areas from 1984 to 2011 in the Cordillera Real, Bolivia.

At a regional scale, a linear increase in both wetland area (+1.15 km^2^/yr, R^2^ = 0.72, p < 0.001) and wetland number (+37.4 wetlands/yr, R^2^ = 0.76, p < 0.001) was evidenced since 1984 (Fig 3). This equals to an estimated increase of 300°% in wetland surface area and 218°% increase in wetland number in 30 years. We also found a strong inter-annual variability in both wetland surface area and number, and a strong positive correlation between these two variables (slope = 3.0, R^2^ = 0.93, p < 0.001; Fig 4A). While the total surface area of wetlands significantly increased over the last 30 years, the distribution of individual wetland size showed no detectable temporal trend in mean and median values. However, we observed a barely significant increase in the top-quartile values of individual wetland surface area over time (slope = 0.8, R^2^ = 0.32, p = 0.076; Fig 4B).

**Fig 5:**
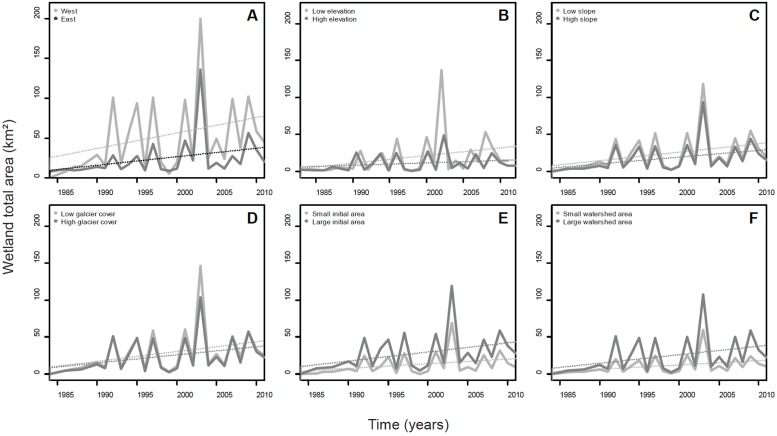
Changes in the total annual high-elevation wetland surface area from 1984 to 2011 in the Cordillera Real, Bolivia. Changes are presented with regards to A) orientation, B) elevation, C) mean slope, D) initial glacier cover in the catchment (in 1984), E) initial wetland surface area, and F) wetland watershed surface area. For B to F, the two time series and trends in each plot refer to small (lower quartile) *vs*. large values (higher quartile) of the environmental variables.

Three environmental variables significantly affected the observed temporal increase in wetland surface area over the last 30 years: orientation, watershed surface area and initial wetland size in 1984 (Fig 5 and Table 1). Surface area expansion was stronger for large wetlands draining large watersheds and located on the western side of the Cordillera. Elevation, slope and the percent of glacier cover in the catchment (% GCC) had no significant effect on the temporal trend in wetland area increase. We found no significant effect on the interaction between time and environmental variables on wetlands surface area trends.

**Table 1:** Results of the analysis of covariance of time and six environmental features (elevation, orientation, slope, watershed surface area, wetland initial size, and glacier cover in the catchment) on the evolution of wetland cover between 1984 and 2014 in the Cordillera Real (Bolivia).

Source	DF	Mean square	F	*P*
Time Elevation Time × elevation Residuals	1 1 1 48	1531 978 352 22033	3.33 2.13 0.77	0.074 0.151 0.385
Time Orientation Time × orientation Residuals	1 1 1 48	8321 12646 722 64763	6.17 9.37 0.53	0.017* 0.003* 0.468
Time Slope Time × slope Residuals	1 1 1 48	3350 448 39 23730	6.78 0.91 0.08	0.012* 0.345 0.778
Time Watershed surface area Time × watershed surface area Residuals	1 1 1 48	2322 2219 288 17429	6.40 6.11 0.79	0.015* 0.017* 0.377
Time Initial size Time × initial surface area Residuals	1 1 1 48	2764 3012 298 19559	6.78 7.39 0.73	0.012* 0.009* 0.396
Time Glacier cover Time × glacier cover Residuals	1 1 1 48	4655 238 33 33986	6.57 0.34 0.05	0.013* 0.565 0.829

* denotes significant effect.

**Fig 6:**
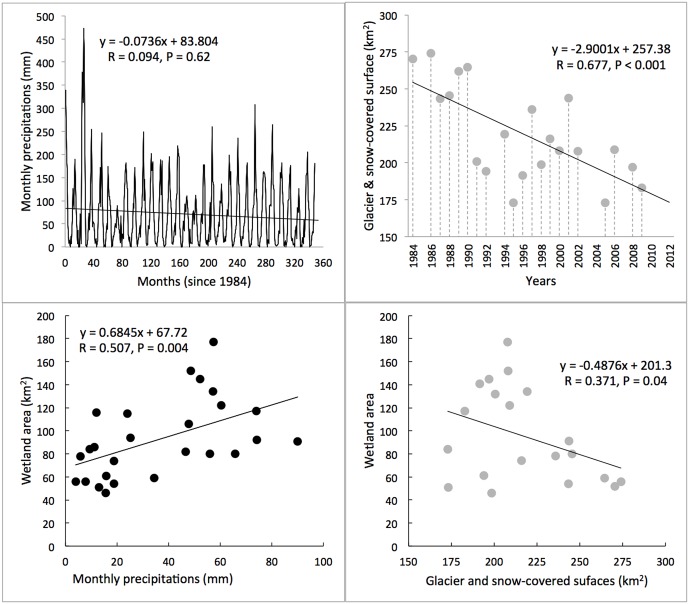
Temporal trends in monthly precipitations (A) and glacier and snow-covered surfaces (B) over the period (1984–2011) in the Cordillera Real, Bolivia. Monthly Precipitation data were obtained from the Zongo valley. Glacier and snow-covered surfaces were measured using LANDSAT images during the dry period (between May and August). Panels C and D give the relationships between wetland cover and precipitation (monthly values over three months prior to the satellite image) and glacier and snow-covered surfaces.

While monthly precipitations showed no significant trends over the (1984–2011) period (Fig 6A), we found a significant decrease in the area of glaciers and snow-covered surfaces over the same period (Fig 6C, p < 0.001). Total wetland area was significantly positively correlated with the monthly precipitation values over the three months preceding the LANDSAT image (Fig 6B, p = 0.004). Glacier and snow-covered surface areas also correlated negatively with total wetland area but the relationship was barely significant (p = 0.04). By combining the two predictors (glacier and snow-covered surfaces, GSC and precipitation, PP) into a multiple regression model, changes in wetland area (W) were predicted by the following equation: W = -0.064 GSC + 1.10 PP + 66.12. The R^2^ value of the two-variable model increased by 0.17 (R^2^ = 0.42) compared to the R^2^ value of the best single variable model.

**Fig 7:**
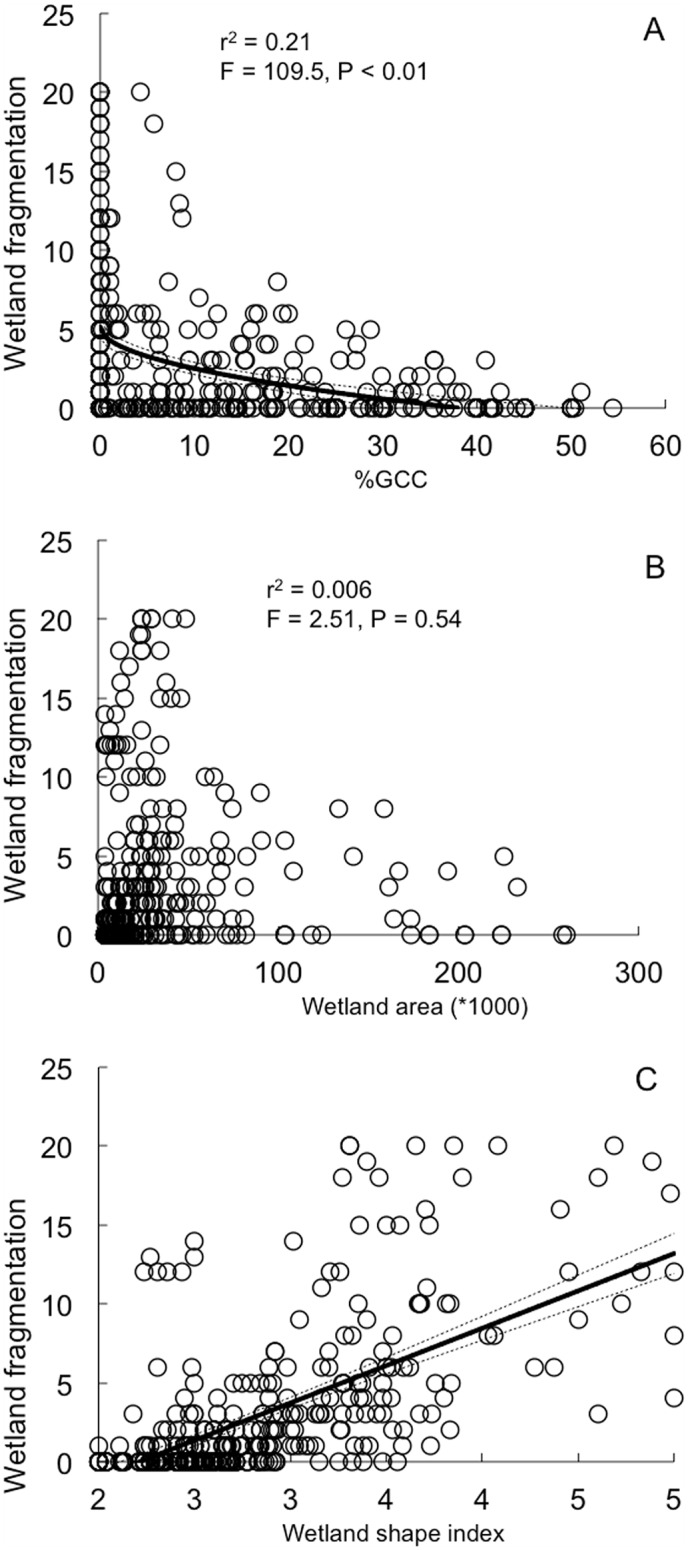
Relationships between wetland drying frequency over the period (1984–2011) and 1984 data and several predictors. % glacier cover in the catchment (A), wetland area (B) and wetland shape index (C), based on 423 wetlands in the Cordillera Real, Bolivia. Data are fitted with linear models. Dotted lines indicate 95% confidence intervals.

**Fig 8:**
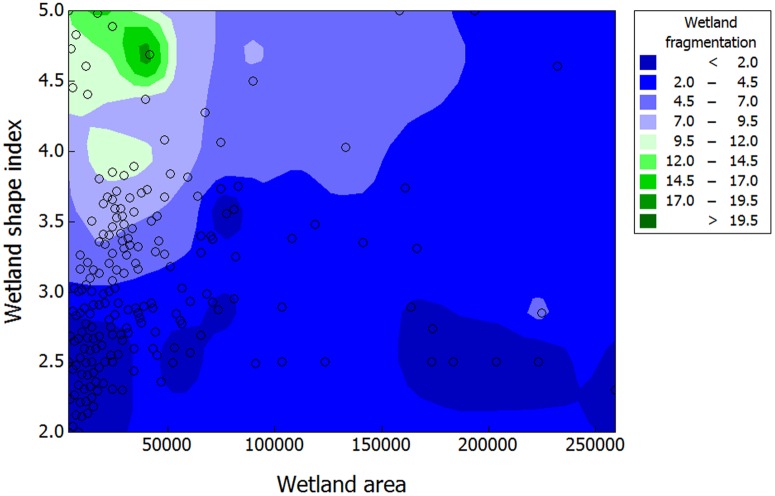
Surface plot of wetland drying index. The index is given as a function of wetland area and wetland shape index (1984 data), based on 423 wetlands in the Cordillera Real, Bolivia.

By following individually a subsample of 423 wetlands, we analyzed wetland-drying frequency as an indicator of wetland vulnerability over the last 30 years. Among the eight variables included in our GLM analysis, %GCC (Likelihood ratio test = 4.7, p = 0.02; Table 2; Fig 7A) and wetland shape index (SI; Likelihood ratio test = 13.1, p < 0.001; Table 2; Fig 7C) were the only single variables that significantly affected wetland drying rate over the last 30 years: round wetlands and those located in highly glacierized catchments were less prone to drying. While the ‘initial area of wetland’ was not significant in the GLM (see Table 2, Fig 7B), its interaction with SI was by far the most significant term affecting wetland drying over the last 30 years (LRT = 48.4, p < 0.0001; Table 2). Relatively small wetlands with high shape index (SI) values, *i*.*e*. irregularly shaped contours were most prone to drying (Fig 8).

**Table 2:** Results of the generalized linear model’s deviance analysis on wetland drying frequency. Only significant interaction effects are shown. AIC is the Akaike’s Information Criterion for the initial model after removal of the ‘‘effect” term. Likelihood-ratio test (LRT) and associated p-value test the hypothesis that the suppression of the ‘‘effect” term provides no better fit than the initial model.

Effect terms	Deviance	AIC	LRT	*P*
Glacier cover in the catchment	1650.9	2305.7	4.7	0.020*
Shape index (SI)	1691.7	2346.4	13.1	< 0.001*
Catchment area	1644.4	2299.2	1.2	0.272
Slope	1643.2	2298.0	0.1	0.944
Elevation	1644.0	2298.7	0.8	0.377
Watershed surface area (WA)	1644.4	2299.2	1.2	0.272
WA × SI	1656.3	2311.0	48.4	< 0.0001*

* denotes significant effect.

**Fig 9:**
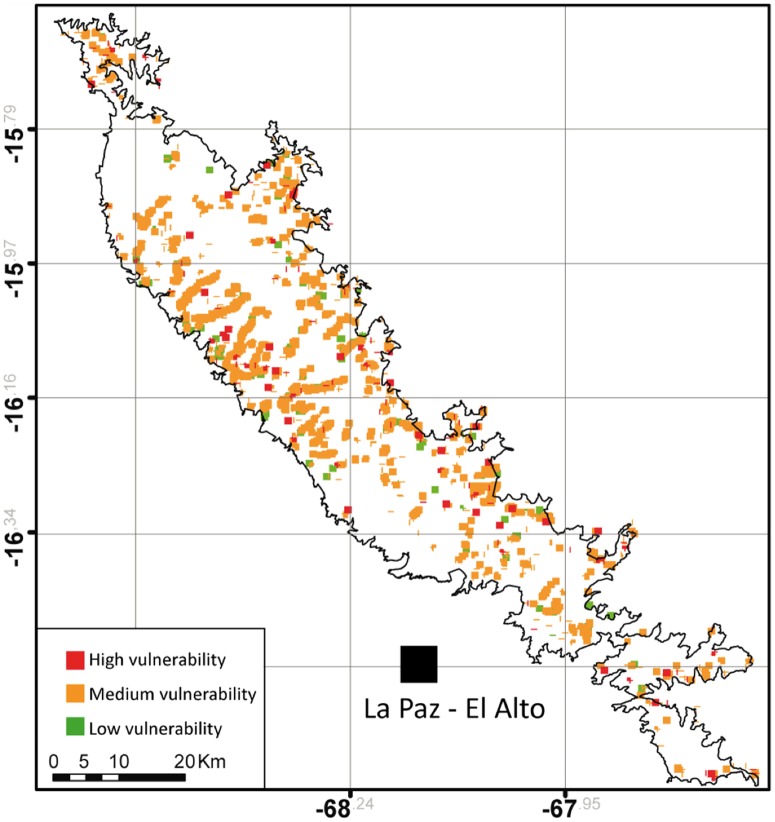
Map of the predictive vulnerability status of 1689 wetlands recorded in 2011 in the Cordillera Real, Bolivia. Wetlands are represented by small marks that define irregular shape where clustered.

Based on results in Fig 7, we built a predictive map of the drying vulnerability for the 1689 wetlands recorded in 2011 the Cordillera Real (Fig 9). Our vulnerability index was calculated as follows: wetland with small area (first quartile of the entire population) and large SI (fourth quartile of the entire population) were considered as highly sensitive to drying and therefore highly vulnerable in the future (red dots, Fig 9). Wetlands with large area (fourth quartile) and low SI (first quartile) had a low vulnerability status (green dots) while the rest of wetlands had an intermediate vulnerability status (orange dots). Of the 1689 wetlands a majority (85.3 %) showed an intermediate vulnerability, 8.5 % showed a high vulnerability and 6.2 % showed a low vulnerability.

## Discussion

### Satellite remote sensing to monitor ecosystem sentinels

Satellite observations have revolutionized our understanding of ecosystem resource monitoring and management [52, 53] and our study is an example of how remote sensing can be used for the monitoring of ecosystem sentinels in the face of climate change. For example, [53] quantified changes in global surface water over the past 32 years at 30-metre resolution, and found a net increase in permanent water, mainly related to reservoir filling and climate change (e.g., glacier melt). Examining the high-resolution map provided by the authors (see https://global-surface-water.appspot.com/) further revealed that wetlands in the Bolivian Cordillera Real showed highly valley-specific trends over the 1984–1999 to 2000–2015 period, ranging from a “decreasing” to “no change” to “increase” in water occurrence intensity. Overall, our findings are in line with the results presented by [53].

Several methodological issues need to be discussed to appreciate potential limitations of our results. For example, the entities outlined in this work, and identified as wetlands, are defined on the basis of a threshold applied on reflectance values of a 900-m^2^ area (surface area of one pixel of a LANDSAT image). This reflectance value characterizes vegetation intensity on that area. It is worth emphasizing that mixed pixels, *i*.*e*. containing both vegetation and bare soil can be missed, as well as entities smaller than 900 m^2^. However, we are confident that our methodology gave a robust estimation of annual changes in the number and surface area of wetlands because 1) all used images have the same spatial resolution, 2) all images were converted from digital number to reflectance values, and 3) the same NDVI threshold was applied to all images. In addition, we already mentioned the relevance of the NDVI to delineate wetlands on LANDSAT images based on the comparison with the high spatial resolution PLEIADES images that allowed a manual delineation of wetlands with a high degree of confidence. Regarding the monitoring of individual wetlands changes, it is worth emphasizing that this monitoring is relative to the initial state of the wetlands, *i*.*e*. the reference year 1984, and that we have no evidence regarding the fact that this year 1984 was peculiar or not.

### Wetland cover trends and climate change

Our work revealed an increase in high-elevation wetland total area over the 1984–2011 period in the Cordillera Real of Bolivia. Changes in wetland cover were positively correlated to precipitation intensities but showed high inter-annual variability. Because precipitation time series did not display any significant trend over the (1984–2011) period it is likely that part of the observed increase in wetland area could be related to an increase frequency in extreme precipitation events over the last decades, a reality that is predicted to accentuate in the future [54]. While we found no relationship between ENSO indices and wetland area or precipitation intensity (results not shown) our data showed longer duration and more intense precipitations events during some negative ENSO years, like 1999–2000 and 2010–2011, in agreement with previous reports in the region [33, 55]. Such intense events coincided with an increased number of LANDSAT cells with high NDVI index, supporting the importance of extreme climatic events in shaping arid and semi-arid ecosystems [56]. In addition to changes in melt water supplies (see below), inter-annual precipitations are likely to play a significant role in wetland cover dynamics but further studies are needed to better understand the underlying mechanism in play. Indeed, changes in wetland cover depend not only on the distribution and intensity of precipitation but also on the land surface properties that control how precipitation is partitioned among evaporation, storage at the surface and at depth, and runoff [57].

Contrastingly to precipitation time series, the time series of glacier and snow-covered surface revealed a significant reduction of about 35 % over the (1984–2011) period. This estimation is in the range of those reported by several, detailed glacier-balance studies performed in the region [19, 58]. Glacier reduction coincided with wetland increase over the study period, and our multiple regression model revealed that the “glacier term” improved the predictive power of the model made by precipitations alone. While correlation does not imply causation, the widely observed increase in ice melt rates in the tropical Andes is expected to a short-term increase in water availability at high elevation [59]. Existing glacier retreat scenarios in the Andes suggest that the increase in water availability will not last and that the observed positive trend in wetland cover is likely to be reversed in a near future [e.g., 60]. Indeed, the current increase in glacier runoff is expected to slow until it reaches its maximum and then start a pronounced decline until the glacier has no more influence on outflows [60]. The probability of this sequence to occur is very high in significantly glacierized watersheds, which is the case for most wetlands in our study area. Also, higher temperatures and excess in water from nearby glacier melt may induce a rapid vegetation growth, a phenomenon that has been evidenced in several landscapes worldwide undergoing an important glacier shrinkage over recent years/decades, e.g., in Chile [61], Alaska [62], and Colombia [63]. In the Cordillera Real, smaller *Distichia* peat areas are found in slopes or plateaus with high runoff or seepage and their formation could be favored during prolonged periods of high runoff (FA and OD, pers. obs.). However, the importance of glacial contribution to runoff is complex as it depends on the magnitude of other components of the hydrological cycle, and is thus regionally highly variable [45, 60].

### Ecological implications of changes in wetland cover and drying

While we found an overall increase in wetland cover over the (1984–2011) period, this trend was not associated with larger areas of individual wetlands but rather to an increase in the total number of wetlands (*i*.*e*. appearance of new, more or less temporary wetlands). As mentioned earlier, our definition of “wetlands” ranges from wet grasslands to peat bogs and it is likely that the documented increase in wetland cover mainly concerned the “wet grassland” category. In contrast with the dominant, cushion-forming species of high Andean wetlands, which require several decades to colonize new habitats in a changing environment [17, 18, 38], species of the Poaceae family have the capacity to perform rapid colonization in alpine systems [64, 65]. In our systems, heavy precipitations and accumulation of water into small depression are expected to rapidly trigger the growth of Poaceae. While these plant assemblages can be consumed by livestock and serve as foraging grounds to wild fauna, they are far from presenting the same economical value than more perennial and complex wetlands like peat bogs, which develop up to 10 m-depth organic soils with exceptionally high water retention and forage for livestock during dry seasons [22, 38]. From a conservation perspective, Poaceae-dominated wetlands have been shown to shelter more generalist species, which are also observed outside wetlands, thus not providing a long-term habitats for the specific biodiversity of cushion-dominated wetlands [39]. Moreover, the monitoring of individual perennial wetland dynamics over the study period clearly demonstrated that they were subject to frequent drying events. These wetlands provide important wildlife habitat in the tropical Andes and the functional connectivity of those habitat patches is crucial for the persistence and resilience of spatially structured wild populations [66] and extensive camelid pastoralism. Wetland drying dynamics was mainly a result of alternating drier and wetter years, a phenomenon well evidenced by the great inter-annual variability in wetland cover trend.

Interestingly, our results suggest that the drying process of wetland habitat can be partly buffered by glacier cover in the wetlands’ catchment, as the drying rate was negatively correlated with percentage of glacier cover in the catchment. This is an important finding in the context of climate change (temperatures are predicted to increase by 5°C over the next 90 years in the Andes, [67, 68]) as many small glaciers whose upper reaches are lower than 5400 m will probably disappear by the middle of the 21^st^ century [19]. Glaciers lying on the highest summits (*i*.*e*. above 6,000 m in the Cordillera Real) will probably lose between 40 and 90% of their volume by 2100 [68]. Taking into account that glacier contribution to water discharge in the Cordillera Real during the dry season can reach close to 27% [21], their shrinkage within the next decades is likely to strongly impact the future of wetland conservation. While glaciers seem to play a significant role in the future of wetland conservation in the Cordillera Real, intrinsic factors such as wetland area and shape index are by far the most reliable variables to predict whether they will be prone to drying or not. This may have strong consequences for biodiversity [17, 39], especially by changing the structure of cushion plant communities, themselves conditioning the presence of other plant species through their important nurse effect [39, 69].

## Conclusion

Despite the correlative nature of our results, wetland cover variations appeared to finely respond to increases in both precipitation extreme events and glacier melting over the 1984–2011 period in the Bolivian Cordillera Real. The observed increase in wetland cover is probably momentary in view of the predicted future decrease in both total precipitations [54] and glacier run-off [21] in the region for the coming decades. Moreover, the increase in wetland surface area and number was mostly due to the apparition of temporary wet grasslands, while more perennial and complex wetlands experienced strong drying processes resulting from alternating drier and wetter years. This type of wetland is at great risk as the presence of rapidly disappearing glaciers in their catchments buffer against drying. High Andean wetlands can therefore be considered as ecosystem sentinel for climate change as they seem sensitive to changes in both extreme rainfall events and glacier melt water supply.

Beyond the specific focus of our study, this work supports the recent view that satellite-based monitoring of ecosystem sentinels can help filling the lack of information on current and changing environmental conditions [70], a common and crucial problem issue in climate change research, particularly in less-developed countries [71]. This opens a promising line of research to better evaluate the conservation status of entire ecosystems, in particular in remote areas. Additional information on the social dynamics associated with these changes are urgently needed as the interaction between the ongoing climate-forced physical changes in land-cover and socio-economic forces affecting land-use has the potential to initiate a destructive positive feedback loop. In the high tropical Andes, grazing, mining, and peat extraction will probably become more intense on the remaining optimal sites as more and more sites become degraded, dooming many of these unique ecosystems and their ability to support livelihoods.

## Supporting information

S1 Fig Calibration between Pléiades and Landsat images. Regression coefficients (red line), slope values (labels on red dots), and root mean-square deviation (RMSE, blue curve) of the linear regressions between wetland areas calculated from Pléiades image and those calculated from Landsat images (N = 33 wetlands at different NDVI threshold values.
(DOCX)

S2 Fig Calculation of wetland fragmentation. Illustration of the different steps of the procedure used to calculate 747 individual wetland fragmentation over the period 1984–2011.
(DOCX)

S1 Table List of LANDSAT images used in this study. The 738 PLEIADES images were 739 recorded the 2013-05-26 and 2013-06-03.
(DOCX)

S1 File Data spreadsheet of wetland cover and main predictors presented in Fig 7. (XLSX)

## References

[1] Bellard C, Bertelsmeier C, Leadley P, Thuiller W, Courchamp F. Impacts of climate change on the future of biodiversity. Ecol Lett. 2012: 15: 365–377. 10.1111/j.1461-0248.2011.01736.x 22257223 PMC3880584

[2] Dawson TP, Jackson ST, House JI, Prentice IC, Mace GM. Beyond predictions: biodiversity conservation in a changing climate. Science 2011; 332: 53–58. 10.1126/science.1200303 21454781

[3] Williamson CE, Brentrup JA, Zhang J, Renwick WH, Hargreaves BR, Knoll LB, et al Lakes as sensors in the landscape: Optical metrics as scalable sentinel responses to climate change. Limnol Oceanogr. 2014; 59: 840–850.

[4] Parmesan C. Ecological and evolutionary responses to recent climate change. Ann Rev Ecol Evol Syst. 2006; 37: 637–669.

[5] Walther GR. Community and ecosystem responses to recent climate change. Phil Trans R Soc B, 2010; 365: 2019–2024. 10.1098/rstb.2010.0021 20513710 PMC2880129

[6] Buytaert W, Cuesta-Camacho F, Tobón C. Potential impacts of climate change on the environmental services of humid tropical alpine regions. Glob. Change Biogeogr. 2011; 20: 19–33.

[7] Salazar LF, Nobre CA, Oyama MD. Climate change consequences on the biome distribution in tropical South America. Geophysl Res Lett. 2007; 34: L09708,

[8] Freeman LA, Kleypas JA, Miller AJ. Coral reef habitat response to climate change scenarios. PLoS ONE, 2013; 8: e82404 10.1371/journal.pone.0082404 24340025 PMC3855618

[9] Hannah DM, Brown LE, Milner AM, Gurnell AM, McGregor GR, Petts GE, et al Integrating climate–hydrology–ecology for alpine river systems. Aquat Cons Mar Freshwat Ecosyst. 2007; 17: 636–656.

[10] Jacobsen D, Cauvy-Fraunié S, Andino P, Espinosa R, Cueva D, Dangles O. Variations in glacial runoff change longitudinal distribution patterns of macroinvertebrates in an Ecuadorian glacier-fed stream: implications for effects of global warming? Freshwat Biol. 2014; 59: 2038–2050.

[11] Schindler DW, Bayley SE, Parker BR, Beaty KG, Cruikshank DR, Fee EJ, Stainton MP. The effects of climatic warming on the properties of boreal lakes and streams at the Experimental Lakes Area, northwestern Ontario. Limnol Oceanogr. 1996; 41: 1004–1017.

[12] Dangles O, Crespo-Pérez V, Andino P, Espinosa R, Calvez R, Jacobsen D. Predicting richness effects on ecosystem function in natural communities: insights from high elevation streams. Ecology 2011; 92: 733–743 21608481 10.1890/10-0329.1

[13] Cannone N, Sgorbati S, Guglielmin M. Unexpected impacts of climate change on alpine vegetation. Front. Ecol. Environ. 2007; 5: 360–364.

[14] Nagy L, Grabherr G. The biology of alpine habitats. Oxford University Press, 2009, 371p.

[15] Grabherr G, Gottfried M, Pauli H. GLORIA: a global observation research initiative in alpine environments. Mount. Res. Dev. 2000; 20: 190–191.

[16] Michelutti N, Wolfe AP, Cooke CA, Hobbs WO, Vuille M, Smol JP. Climate change forces new ecological states in tropical Andean lakes. PLoS ONE 2015; 10: e0115338 10.1371/journal.pone.0115338 25647018 PMC4315470

[17] Squeo FA, Warner BG, Aravena R, Espinoza D. Bofedales: high altitude peatlands of the central Andes. Rev Chil Hist Nat. 2006; 79: 245–255.

[18] Anthelme F, Cavieres L.A., Dangles O. Facilitation among plants in alpine environments in the face of climate change. Front Plant Sci, 2014; 5: 1–15 10.3389/fpls.2014.00387 PMC4130109 25161660

[19] Rabatel A, Francou B, Soruco A, Gomez J, Cáceres B, Ceballos J et al Current state of glaciers in the tropical Andes: a multi-century perspective on glacier evolution and climate change, Cryosphere 2013; 7: 81–102.

[20] Colwell RK, Brehm G, Cardelús CL, Gilman AC, Longino JT. Global warming, elevational range shifts, and lowland biotic attrition in the wet tropics. Science 2008; 322: 258–261. 10.1126/science.1162547 18845754

[21] Soruco A, Vincent C, Rabatel A, Francou B, Thibert E, Sicart JE, Condom T. Impacts of glacier shrinkage on water resources of La Paz city, Bolivia (16°S). Ann Glaciol, 2015; 56: 147–154.

[22] Cooper DJ, Wolf EC, Colson C, Vering W, Granda A, Meyer M. Alpine peatlands of the Andes, Cajamarca, Peru. Arctic Antarctic Alpine Res. 2010; 42: 19–33.

[23] Wieder RK Past, present, and future peatland carbon balance: An empirical model based on 210Pb-dated cores. Ecol Appl. 2001; 11: 327–342.

[24] Delarue F. Persistent high temperature and low precipitation reduce peat carbon accumulation. Glob. Change Biol., 2016; 22: 3253–3254. 10.1111/gcb.13433 27414237

[25] Bragazza L, Buttler A, Robroek BJM, Albrecht R, Zaccone C, Jassey VEJ, Signarbieux C Persistent high temperature and low precipitation reduce peat carbon accumulation. Glob. Change Biol. 2016; 22: 4114–4123. 10.1111/gcb.13319 27081764

[26] Anderson E, Marengo J, Villalba R, Halloy S, Young B, Cordero D, et al Consequences of climate change for ecosystems and ecosystem services in the tropical Andes In Herzog SK, Martínez R, Jørgensen PM, Tiessen H, editors. Climate Change and Biodiversity in the Tropical Andes. Instituto Interamericano para la Investigación del Cambio Global, 2011 pp. 1–18.

[27] Belyea LR, Malmer N. Carbon sequestration in peatland: patterns and mechanisms of response to climate change. Glob Change Biol. 2004; 10: 1043–1052.

[28] Buttolph LP, Coppock DL. Influence of deferred grazing on vegetation dynamics and livestock productivity in an Andean pastoral system. J. Appl. Ecol. 2004; 41: 664–674.

[29] Food and Agriculture Organization (2007) FAOSTAT. Retrieved February 25, 2015 (http://faostat.fao.org/).

[30] Otto M, Scherer D, Richters J. Hydrological differentiation and spatial distribution of high altitude wetlands in a semi-arid Andean region derived from satellite data. Hydrol Earth Sci Syst. 2011; 15: 1713–1727.

[31] Rabatel A, Bermejo A, Loarte E, Soruco A, Gomez J, Leonardini G, Vincent C, Sicart JE. Can the snowline be used as an indicator of the equilibrium line and mass balance for glaciers in the outer tropics? J Glaciol. 2012; 58: 1027–1036.

[32] Garreaud R, Vuille M, Clement A. The climate of the Altiplano: observed current conditions and mechanisms of past changes. Palaeogeogr Palaeoclim Palaeoecol 2003; 194: 5–22.

[33] Ronchail J, Gallaire R. ENSO and rainfall along the Zongo valley (Bolivia) from the Altiplano to the Amazon basin. Int J Climatol. 2006; 26: 1223–1236.

[34] Vuille M, Francou B, Wagnon P, Juen I, Kaser G, Mark BG, Bradley RS. Climate change and tropical Andean glaciers: Past, present and future, Earth Sci Rev. 2008; 89, 79–96.

[35] Vuille M, Bradley RS, Werner M, Keimig F. 20th century climate change in the tropical Andes: observations and model results, Clim Change, 2003; 59: 75–99.

[36] Haylock MR, Peterson TC, Alves LM, Ambrizzi T, Anunciacao MT, Baez J et al Trends in total and extreme South American rainfall in 1960–2000 and links with sea surface temperature. J Climatol. 2006; 19: 1490–1512.

[37] Seiler C, Hutjes RWA, Kabat P. Likely ranges of climate change in Bolivia. J Appl Meteorol Climatol. 2013; 52: 1303–1317.

[38] Ruthsatz B. Vegetación y ecología de los bofedales altoandinos de Bolivia. Phytocoenologia 2012, 42: 133–179.

[39] Loza Herrera S, Meneses R, Anthelme F. Comunidades vegetales de los bofedales de la Cordillera Real (Bolivia) bajo el calentamiento global. Ecol Bol. 2015; 50: 39–56.

[40] Tellería JL, Venero JL, Santos T. Conserving birdlife of Peruvian highland bogs: effects of patch-size and habitat quality on species richness and bird numbers. Ardeola 2006; 53: 271–83.

[41] Anthelme F, Meneses RI, Dangles O Editors. Métodos para estudiar el efecto del cambio climático sobre los bofedales y sus servicios ambientales inherentes. Ecol Bol 2014; 49: 1–154.

[42] Quenta E, Molina-Rodriguez J, Gonzales K, Rebaudo F, Casas J, Jacobsen D, Dangles O. Glacier influence on aquatic biodiversity in high Andean peatlands. Glob Change Biol. 2016; 22: 3196–3205. 10.1111/gcb.13310 27058991

[43] Gardner RC, Davidson NC. The Ramsar Convention In LePage BA Editor, Wetlands. Springer Netherlands, 2011; 189–203.

[44] Hall DK, Riggs GA, Salomonson VV. Development of methods for mapping global snow cover using moderate resolution imaging spectroradiometer data. Remote Sens. Environ. 1995; 54: 127–140.

[45] Cauvy-Fraunié S, Condom T, Rabatel A, Villacis M, Jacobsen D, Dangles O. Using wavelet analyses on water depth time series to detect glacial influence in high-mountain hydrosystems, Hydrol. Earth Syst. Sci. 2013; 17: 4803–4816.

[46] Matthews KR, Pope KL. A telemetric study of the movement patterns and habitat use of *Rana muscosa*, the mountain yellow-legged frog, in a high-elevation basin in Kings Canyon National Park, California. J Herpetol. 1999; 33: 615–624.

[47] Krämer B, Poniatowski D, Fartmann T. Effects of landscape and habitat quality on butterfly communities in pre-alpine calcareous grasslands. Biol Cons. 2012; 152: 253–261.

[48] Declerck SAJ, Coronel JS, Legendre P, Brendonck L. Scale dependency of processes structuring metacommunities of cladocerans in temporary pools of High-Andes wetlands. Ecography 2011; 34: 296–305.

[49] Wang X, Blanchet FG, Koper N. Measuring habitat fragmentation: an evaluation of landscape pattern metrics. Meth Ecol Evol. 2014; 5: 634–646.

[50] Kupfer JA. Landscape ecology and biogeography. Progr- Phys. Geogr. 1995; 19: 18–34.

[51] Cochrane MA, Laurance WF. Fire as a large-scale edge effect in Amazonian forests. J. Trop. Ecol. 2002; 18: 311–325.

[52] Famiglietti JS, Cazenave A, Eicker A, Reager JT, Rodell M, Velicogna I. Satellites provide the big picture. Science 2015; 349: 684 10.1126/science.aac9238 26273037

[53] Pekel JF, Cottam A, Gorelick N, Belward AS. High-resolution mapping of global surface water and its long-term changes. Nature 2016; 540: 418–422. 10.1038/nature20584 27926733

[54] Thibeault JM, Seth A, Garcia M. Changing climate in the Bolivian Altiplano: CMIP3 projections for temperature and precipitation extremes. J Geophys Res, 2010; 115(D8).

[55] Bookhagen B, Strecker MR. Modern Andean rainfall variation during ENSO cycles and its impact on the Amazon drainage basin Amazonia, landscape and species evolution: a look into the past, 1^st^ ed, Blackwell, Oxford, 2010; pp 223–243.

[56] Holmgren M, Stapp, Dickman CR, Gracia C, Graham S, Gutiérrez JR. Extreme climatic events shape arid and semiarid ecosystems. Front Ecol Environ. 2006; 4: 87–95.

[57] Prigent C, Papa F, Aires F, Rossow WB, Matthews E. Global inundation dynamics inferred from multiple satellite observations, 1993–2000. J Geophys Res. 2007; 112: D12107.

[58] Soruco A, Vincent C, Francou B, Gonzalez JF Glacier decline between 1963 and 2006 in the Cordillera Real, Bolivia. Geophys Res Lett. 2009; 36(3).

[59] Vuille M. Climate Change and Water Resources in the Tropical Andes. Inter-American Development Bank Environmental Safeguards Unit Technical Note, 2013; No. IDB-TN-515.

[60] Baraer M, Mark BG, McKenzie JM, Condom T, Bury J, Huh K et al Glacier recession and water resources in Peru’s Cordillera Blanca. J Glaciol. 2012; 58: 134–150.

[61] Earle LR, Warner BG, Aravena R. Rapid development of an unusual peat-accumulating ecosystem in the Chilean Altiplano. Quater Res. 2002; 59: 2–11.

[62] Jones M, Yu ZC. Rapid deglacial and early Holocene expansion of peatlands in Alaska. PNAS, 2010; 107: 7347–7352. 10.1073/pnas.0911387107 20368451 PMC2867679

[63] Benavides JC, Vitt DH, Wieder RK. The influence of climate change on recent peat accumulation patterns of *Distichia muscoides* cushion bogs in the high-elevation tropical Andes of Colombia. J Geophys Res Biogeosci. 2013; 118: 1627–1635.

[64] Matthews JA. The Ecology of Recently-deglaciated Terrain: A Geoecological Approach to Glacier Forelands. 1992; Cambridge University Press.

[65] Erschbamer B. Winners and losers of climate change in a central alpine glacier foreland. Arctic Antarctic Alpine Res. 2007; 39: 237–244.

[66] Uden DR, Hellman ML, Angeler DG, Allen CR. The role of reserves and anthropogenic habitats for functional connectivity and resilience of ephemeral wetlands. Ecol Appl. 2014; 24: 1569–1582. 29210223 10.1890/13-1755.1

[67] Bradley RS, Vuille M, Diaz HF, Vergara W. Threats to water supplies in the Tropical Andes. Science 2006, 312, 1755–1756. 10.1126/science.1128087 16794068

[68] Réveillet M, Rabatel A, Gillet-Chaulet F, Soruco A. Simulations of changes in Glaciar Zongo (Bolivia, 16°S) over the 21st century using a 3D full-Stokes model and CMIP5 climate projections. Ann Glaciol, 2015; 56: 89–97.

[69] Anthelme F, Dangles O. Plant–plant interactions in tropical alpine environments. Persp Plant Ecol Evol Sys 2012; 14: 363–372.

[70] Maron M, Gordon A, Mackey BG. Agree on biodiversity metrics to track from space. Nature 2015; 523: 403–405. 10.1038/523403a 26201582

[71] Williamson CE, Saros JE, Schindler DW. Sentinels of change. Science, 2009; 323: 887–888. 10.1126/science.1169443 19213905

