# Supplementary material for: Ecosystem sentinels for climate change? Evidence of wetland cover changes over the last 30 years in the tropical Andes
Source: PLoS One. 2017 May 24;12(5):e0175814. doi: 10.1371/journal.pone.0175814 (PMC5443494; doi:10.1371/journal.pone.0175814)
Supplement: S1 Fig — Regression coefficients (red line), slope values (labels on red dots), and root mean-square deviation (RMSE, blue curve) of the linear regressions between wetland areas calculated from Pléiades image and those calculated from Landsat images (N = 33 wetlands at different NDVI threshold values. (DOCX) [file pone.0175814.s001.docx]

**Figure S1.** Regression coefficients (red line), slope values (labels on red dots), and root-mean-square deviation (RMSE, blue curve) of the linear regressions between wetland areas calculated from Pléiades image and those calculated from Landsat images (N = 33 wetlands) at different NDVI threshold values.

***
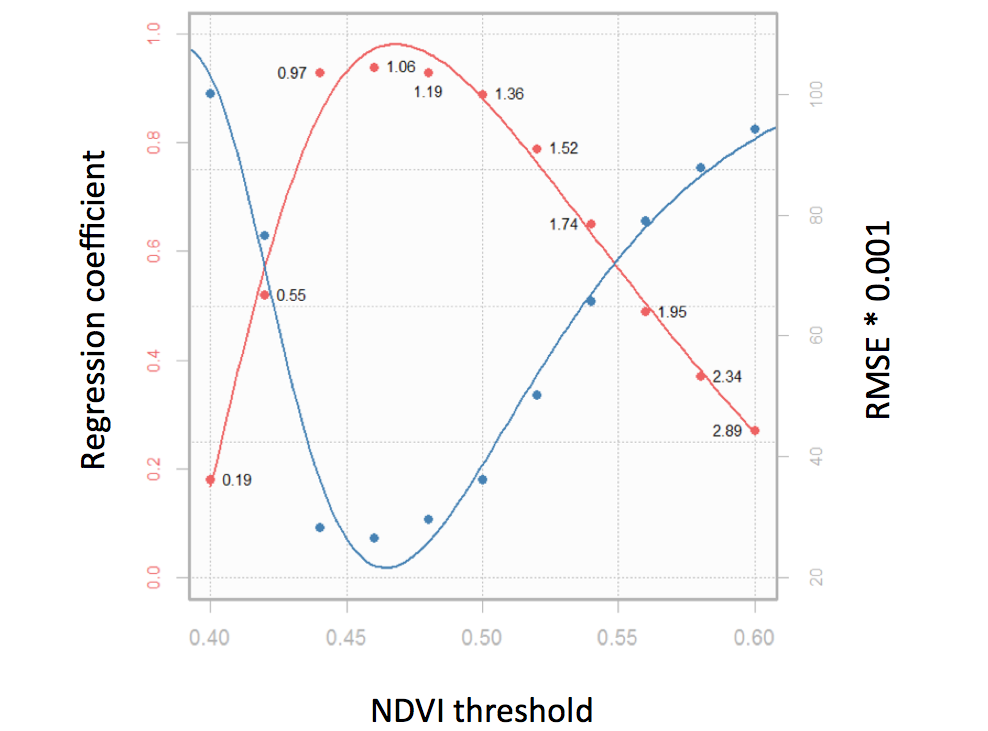
***
