# Supplementary material for: Ecosystem sentinels for climate change? Evidence of wetland cover changes over the last 30 years in the tropical Andes
Source: PLoS One. 2017 May 24;12(5):e0175814. doi: 10.1371/journal.pone.0175814 (PMC5443494; doi:10.1371/journal.pone.0175814)

**Figure S2.** Illustration of the procedure used to calculate individual wetland drying frequency and intensity over the period 1984-2011.


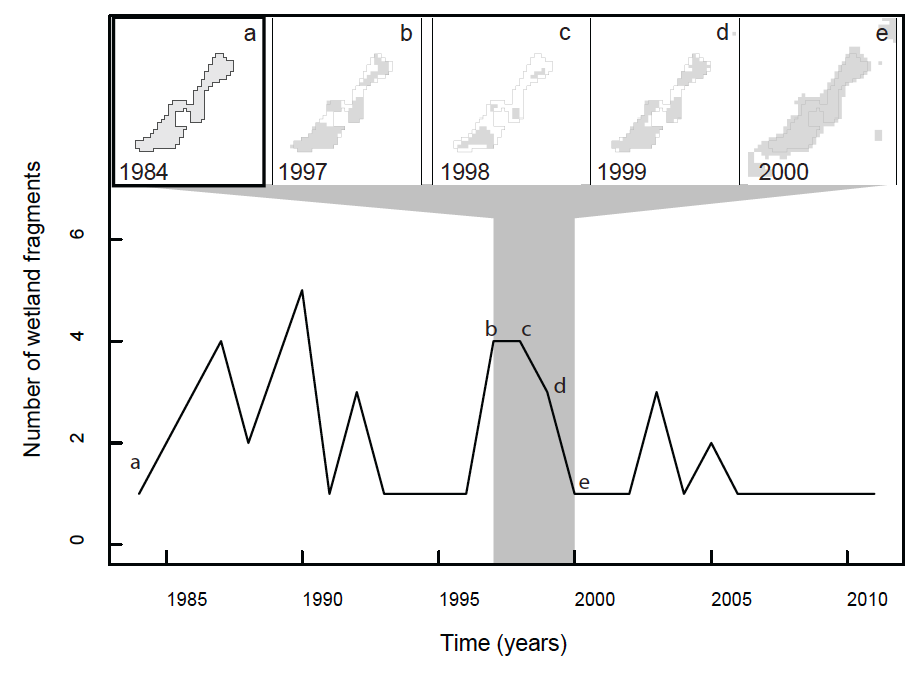

Supplement: S2 Fig — Illustration of the different steps of the procedure used to calculate 747 individual wetland fragmentation over the period 1984–2011. (DOCX) [file pone.0175814.s002.docx]
