# Supplementary material for: Ecosystem sentinels for climate change? Evidence of wetland cover changes over the last 30 years in the tropical Andes
Source: PLoS One. 2017 May 24;12(5):e0175814. doi: 10.1371/journal.pone.0175814 (PMC5443494; doi:10.1371/journal.pone.0175814)
Supplement: S1 Table — The 738 PLEIADES images were 739 recorded the 2013-05-26 and 2013-06-03. (DOCX) [file pone.0175814.s003.docx]

**Table S1.** List of LANDSAT images used in this study. The PLEIADES images were recorded the 2013-05-26 and 2013-06-03.

| Satellite | Scene | Year | Month | Day |
| --- | --- | --- | --- | --- |
| Landsat-5 | LT50010711984190AAA04 | 1984 | 07 | 08 |
| Landsat-5 | LT50010711986195XXX02 | 1986 | 05 | 14 |
| Landsat-5 | LT50010711987214XXX07 | 1987 | 08 | 02 |
| Landsat-5 | LT50010711988185CUB00 | 1988 | 05 | 03 |
| Landsat-5 | LT50010711990206CUB00 | 1990 | 07 | 25 |
| Landsat-5 | LT50010711991145CUB01 | 1991 | 05 | 25 |
| Landsat-5 | LT50010711992148CUB00 | 1992 | 05 | 27 |
| Landsat-5 | LT50010711993150CUB00 | 1993 | 05 | 30 |
| Landsat-5 | LT50010711994153CUB00 | 1994 | 06 | 02 |
| Landsat-5 | LT50010711995172CUB00 | 1995 | 06 | 21 |
| Landsat-5 | LT50010711996143CUB00 | 1996 | 05 | 22 |
| Landsat-5 | LT50010711997193CUB00 | 1997 | 07 | 12 |
| Landsat-5 | LT50010711998212COA03 | 1998 | 07 | 31 |
| Landsat-5 | LT50010711999183CUB04 | 1999 | 07 | 02 |
| Landsat-7 | LE70010712000146EDC00 | 2000 | 05 | 25 |
| Landsat-7 | LE70010712001180CUB01 | 2001 | 06 | 29 |
| Landsat-7 | LE70010712002135COA00 | 2002 | 05 | 15 |
| Landsat-5 | LT50010712003178CUB00 | 2003 | 06 | 27 |
| Landsat-5 | LT50010712004149COA01 | 2004 | 05 | 28 |
| Landsat-5 | LT50010712005167COA01 | 2005 | 06 | 16 |
| Landsat-5 | LT50010712006138COA01 | 2006 | 05 | 18 |
| Landsat-5 | LT50010712007141COA00 | 2007 | 05 | 21 |
| Landsat-5 | LT50010712008128COA00 | 2008 | 05 | 07 |
| Landsat-5 | LT50010712009146COA01 | 2009 | 05 | 26 |
| Landsat-5 | LT50010712010165CUB00 | 2010 | 06 | 14 |
| Landsat-5 | LT50010712011136CUB00 | 2011 | 05 | 16 |
